# The Emergence of Carbon Nanomaterials as Effective Nano-Avenues to Fight against COVID-19

**DOI:** 10.3390/ma16031068

**Published:** 2023-01-25

**Authors:** Joydip Sengupta, Chaudhery Mustansar Hussain

**Affiliations:** 1 Department of Electronic Science, Jogesh Chandra Chaudhuri College, Kolkata 700033, India; 2 Department of Chemistry and Environmental Science, New Jersey Institute of Technology, Newark, NJ 07102, USA

**Keywords:** SARS-CoV-2, carbon nanomaterials, antiviral, biosensor, vaccine

## Abstract

COVID-19 (Coronavirus Disease 2019), a viral respiratory ailment that was first identified in Wuhan, China, in 2019, and then expanded globally, was caused by Severe Acute Respiratory Syndrome Coronavirus 2 (SARS-CoV-2). The severity of the illness necessitated quick action to cease the virus’s spread. The best practices to avert the infection include early detection, the use of protective clothing, the consumption of antiviral medicines, and finally the immunization of the patients through vaccination. The family of carbon nanomaterials, which includes graphene, fullerene, carbon nanotube (CNT), and carbon dot (CD), has a great deal of potential to effectively contribute to each of the main trails in the battle against the coronavirus. Consequently, the recent advances in the application of carbon nanomaterials for containing and combating the SARS-CoV-2 virus are discussed herein, along with their associated challenges and futuristic applicability.

## 1. Introduction

**Figure 1:**
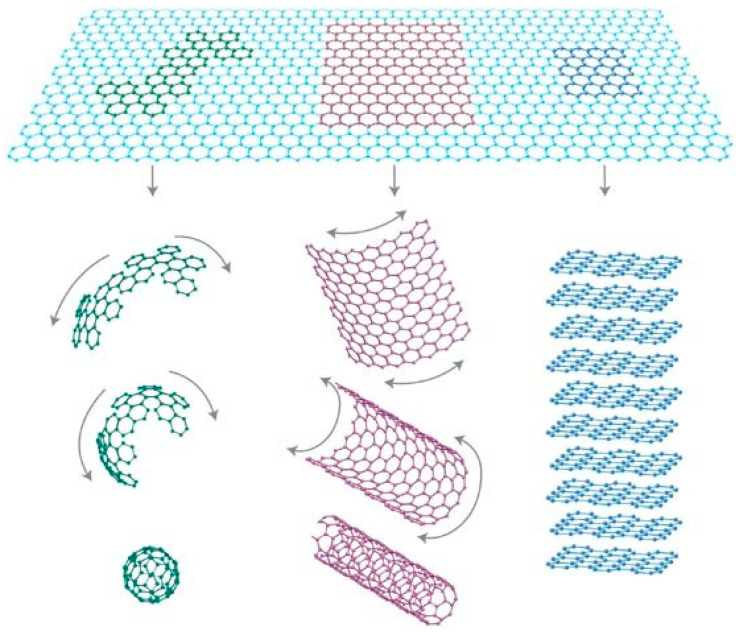
Graphene is a 2D material that acts as a building block for carbonaceous materials of all other dimensions. (Reproduced with permission from Ref. [3]).

Carbon nanomaterials can be categorized based on their dimensionalities, such as 0D (fullerene and CD), 1D (CNT), and 2D (graphene) (Figure 1). Each category of carbon nanomaterials exhibits unique qualities and is utilized in a variety of fields [1], due to their superior physical and chemical characteristics such as high mechanical strength, large electrical conductivity, huge electrochemical sensitivity, adequate efficacy against germs and viruses, excellent surface-to-volume ratio, minimal weight, etc. [2].

Pathogens have repeatedly proven to be lethal to humans throughout the history of mankind [4].

COVID-19, also known as the coronavirus disease, is a highly contagious and potentially fatal illness caused by the SARS-CoV-2 virus. It was first identified in Wuhan, China, in 2019 [5] and quickly spread to become a global pandemic. The virus primarily spreads through respiratory droplets, and symptoms include fever, cough, and difficulty breathing. The impact of COVID-19 has been significant and far-reaching, affecting nearly every aspect of society. The most obvious impact has been on public health, with millions of people becoming infected and hundreds of thousands dying worldwide [6]. The virus has also had a major economic impact, leading to widespread job losses, business closures, and economic downturns [7]. Additionally, the pandemic has led to disruptions in education, travel, and other areas of daily life. Efforts to defeat COVID-19 have been ongoing since the start of the pandemic. These efforts include the development and distribution of vaccines, the implementation of social distancing and other mitigation measures, and the ramping up of testing and contact tracing. The World Health Organization (WHO) and other international organizations have been working to coordinate the global response to the pandemic. Many countries have also implemented measures to try to control the spread of the virus. The vaccines that have been developed for COVID-19 are critical in the fight against the disease [8]. They were highly effective in preventing severe illness and death from COVID-19 and have been authorized for emergency use by many countries around the world. However, the situation is still evolving, and it is important to note that even with the vaccines, the virus is still spreading [9]. So it is crucial to continue following public health guidelines, including wearing masks, practicing physical distancing, and getting tested if anyone has symptoms; the motto continues to be “identification and isolation”.

Since the SARS-CoV-2 virus’s discovery, scientists all over the world have been engaged in extensive research to realize appropriate materials to control and combat the disease employing state-of-the-art technology [10]. Until now, nanotechnology has provided the most efficient means of confining and eliminating viruses such as COVID-19. Using nanotechnology, nanomaterials can be prepared and used to build intelligent nanostructures to inhibit viral proliferation [11]. Among different nanomaterials, carbon nanomaterials are promising candidates for fighting against COVID-19 because of their nanoscale nature, high optical transparency, sufficient biocompatibility, and supreme magnetic properties, and they played a pivotal role in combatting COVID-19 [12,13,14,15]. The different forms of carbon nanomaterials are meticulously employed for the inhibition of COVID-19 in various sectors such as biosensors, personal protective equipment (PPE), antiviral agents, and vaccines; these have been reviewed here with the pros and cons.

## 2. Applications of Carbon Nanomaterials against COVID-19

### 2.1. Carbon Nanomaterials as Sensor for Detection of SARS-CoV-2

“Break the Chain” was the motto of people around the world to stop the rapid spread of COVID-19. The best way to achieve this goal is to diagnose the affected people and isolate them. Biosensors played a critical role in breaking the transmission chain, nanomaterials were proposed to be their key sensing element [16], and functionalization plays a major role in enhancing their sensing efficiency [17]. However, perception of property-performance connections and the optimization process is necessary to fabricate biosensors for accurate and rapid point-of-care (POC) diagnosis.

Aggregation-induced emission carbon dots were found to be one of the most promising fluorescent probe materials because they display excellent emission efficiency in both aggregate and solid forms [18]. This phenomenon was explored by Ju et al. [19] to construct a unique fluorescent lateral flow immunoassay (LFA) platform for the identification of SARS-CoV-2-specific IgM and IgG. The carbodiimide process coupled the SARS-CoV-2 S protein with aggregation-induced emission carbon dots to create the fluorescent complex. The sandwich structure “antigen-antibody-anti-antibody” was used to detect antibodies, while fluorescent immunochromatographic test strips were employed for the concurrent and independent detection of SARS-CoV-2 S IgM and IgG with a LOD of 100 pg/ml. Fluorescence-based molecular level detection of viral RNA of coronavirus at the early stage of infection was also achieved by employing carbon dots [20]. The biorecognition components (antisense oligonucleotides (ASO)) were applied to the surface of carbon dots by the researchers. They observed that the functionalization helped to improve the measured fluorescence through aggregation-induced emission enhancement (AIEE), after hybridization with SARS-CoV-2 RNA with a LOD of 81 copies per μL. Xu et al. [21] developed carbon-dot-based silica spheres for the rapid detection of SARS-CoV-2 nucleocapsid proteins. The N-(aminoethyl)-aminopropyltrimethoxy molecule has two ends, one of which is embedded in the silica carrier and the other of which has amino endings that anchor carboxyl-rich carbon dots and boost the red emission. The composite silica spheres were subsequently coated with 3-(triethylsilyl) propylamine to safeguard the carbon dots, encourage bioconjugation, and produce RCS spheres. This assay demonstrated the benefits of high specificity and straightforward operation and exhibited a LOD of 10 pg mL^−1^.

**Figure 2:**
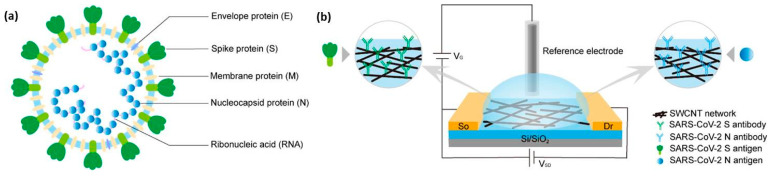
The detection of SARS-CoV-2 Ag using SWCNT-based FET biosensors. (**a**) Schematic structure of SARS-CoV-2 to demonstrate the targeting proteins. (**b**) Schematic illustration of a liquid-gated SWCNT FET for the detection of SARS-CoV-2 SAg and NAg. Interdigitated gold electrodes (yellow blocks) are configured as the source (So) and drain (Dr). (Reproduced with permission from Ref. [26]).

Kim et al. [22] reported that lower surface roughness and precise CNT alignment could result in improved sensitivity at a given film thickness, which could be accounted for by an increased surface area for bio-receptor binding and a reduction in the tube-to-tube connection density. They created immunosensors for detecting the SARS-CoV-2 virus, employing two types of CNT thin films: High Alignment Low Roughness (HALR) and Low Alignment High Roughness (LAHR). In terms of limit of detection (LOD), the HALR- and LAHR-based sensors had values of 5.62 and 10.59 fg/mL, respectively. Field effect transistor (FET) type sensors were fabricated by different groups and used carbon nanomaterials as the key channel element to detect SARS-CoV-2 [23]. A fast, user-friendly, inexpensive, sensitive, and selective electrochemical biosensor employing a p-channel depletion-type CNT-FET structure was fabricated by Zamzami et al. [24]. CNTs were screen-printed on a Si/SiO_2_ surface to act as a channel, and the SARS-CoV-2 S1 antibody was immobilized on CNTs by a non-covalent interaction with a linker. The sensor was able to detect the SARS-CoV-2 S1 antigens in fortified saliva samples with high reproducibility by monitoring the change in channel conductance with a LOD of 4.12 fg/mL. The flexible Kapton substrate was used by Thanihaichelvan et al. [25] to fabricate the liquid-gated CNT-FET, which served as the signal transducer, while the RNA hybridization onto the CNT channel acted as the main signal generator. The biosensor responded selectively to the positive target sequence, with a LOD of 10 fM. Antibody-functionalized (either anti-SARS-CoV-2 spike protein antibody (SAb) or anti-nucleocapsid protein antibody (NAb)) CNT-FET biosensors were developed by Shao et al. [26] to detect the novel coronavirus antigen in clinical nasopharyngeal samples without prior sample processing (Figure 2). The high analytical sensitivity and specificity were proven by the low LOD values of 0.55 fg/mL for SAg and 0.016 fg/mL for NAg.

The proper extraction of viral single-stranded nucleic acids is a vital process for the faithful detection of the coronavirus. Jeong et al. [27] developed a high-yield, facile route to extract viral single-stranded nucleic acids by employing “capture” ssDNA sequences that were attached to CNTs. The CNT-based capturing process outperforms the extraction yield of commercial silica-column kits and commercial DNA/RNA extraction kits. This method also increased the identification sensitivity of viral nucleic acids for coronavirus detection using quantitative reverse transcription polymerase chain reaction (RT-qPCR). For the respiration monitoring of patients affected by SARS-CoV-2, a biodegradable and flexible breath sensor was developed by Thiyagarajan et al. [28] using a paper substrate to exploit its hygroscopic nature, and the sensor revealed a high sensitivity of almost 8.24 pF/Rh% for humidity levels above 70%. The researchers screen-printed CNTs-polydimethylsiloxane (PDMS) composite-based inter-digitated electrodes on a paper substrate, and during the experiment, the capacitance of the paper changed with the alteration in humidity during inhalation and exhalation. For rapid coronavirus detection, Pinals et al. [29] developed an optical CNT-based nanosensor. The CNTs were non-covalently functionalized with a protein called angiotensin-converting enzyme 2 (ACE2), which has a high binding affinity for the SARS-CoV-2 spike protein. In the presence of SARS-CoV-2, the fluorescence intensity of the nanosensor doubled itself within 90 min to facilitate faithful detection of the virus with a limit of 12.6 nM. For the initial screening of patients infected with SARS-CoV-2, Hasan et al. [30] prepared a CNT-based on-body patch antenna for detecting the condition of COVID-19-affected lungs. To develop the antenna, a primarily CNT solution was spin-coated on a flame-retardant 4 (FR4) substrate, and then the antenna was operated in the range of 6.63–7.29 GHz with a maximum specific absorption rate of 1.77 W/kg for 10 g of tissues. By attaching the SARS-CoV-2 spike protein to carbon-based screen-printed electrodes (SPEs), Cardoso et al. [31] created an ultra-sensitive biosensor that allowed for thorough SARS-CoV-2 antibody capture. To increase conductivity, carboxylated CNTs were bonded to SPE, and EDC/NHS chemistry was later used to create an amine layer that could sensitively detect SARS-CoV-2 antibodies with a LOD value of 0.7 pg/mL. Curti et al. [32] fabricated a CNT-SPE-based single-step, reagent-less electrochemical sensing platform for the detection of SARS-CoV-2 spike protein with a LOD of 7 nM. In the presence of the virus, a redox-tagged DNA aptamer that was coupled with SPE gets folded, reducing the current flowing between the redox tag and the electrode surface and enabling detection. Li et al. [33] constructed an immunoresistive sensor for the detection of both the alpha and delta forms of SARS-CoV-2 in nasal swab samples using monoclonal antibody-functionalized CNT. To reduce the contact resistance, silver electrodes were silkscreened-printed on CNTs before the biosensor was constructed on a polyethylene terephthalate (PET) film. The resistance ratio between the SARS-CoV-2 sensor electrodes and the control electrodes was assessed using a LOD value of 350 genome equivalents/mL to boost the detection methodology’s sensitivity and specificity. An avidin-functionalized CNT-based bio-capture technology was used by Mujica et al. [34] to develop impedimetric as well as amperometric genosensors for sensitive measurement of SARS-CoV-2 nucleic acid. The biotinylated DNA probes were immobilized on glassy carbon electrodes (GCE), employing avidin-functionalized CNT. SARS-CoV-2 nucleic acid was quantified at two different scales: aM and zM, using the impedimetric biosensor and the amperometric biosensor, respectively. To imprint the whole SARS-CoV-2 particles inside the polymeric matrix and produce viral complementary binding sites, an electrochemical sensing platform was developed by Hussein et al. [35] employing CNTs/WO_3_-SPE. Different viral concentrations were used to assess the sensor chips’ sensitivity performance at a LOD of 57 pg/mL.

**Figure 3:**
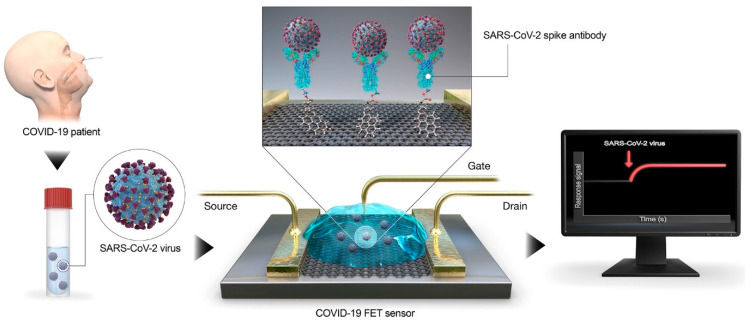
Schematic diagram of the COVID-19 FET sensor operation procedure. Graphene as a sensing material is selected, and the SARS-CoV-2 spike antibody is conjugated onto the graphene sheet via the 1-pyrenebutyric acid N-hydroxysuccinimide ester, which is an interfacing molecule as a probe linker. (Reproduced with permission from Ref. [37]).

For effective sensing of a virus, selection of a proper biomarker is crucial, and heptanal was found to be a critical biomarker for identifying SARS-CoV-2. [36]. Density functional theory was used to analyze the adsorption characteristics of heptanal on both undoped graphene and graphene doped with transition metals (Pd, Pt, and Ag). Doped graphene’s electronic properties changed dramatically after heptanal adsorption when compared to pristine graphene, while Ag-doped graphene revealed the most promising results. To detect coronavirus in clinical specimens, Seo et al. [37] developed a graphene-based FET (GFET) by conjugating the SARS-CoV-2 spike antibody to the graphene sheet employing an interface coupling agent (Figure 3). The SARS-CoV-2 virus in clinical samples, the SARS-CoV-2 antigen in common buffer and transport medium, and the cultured SARS-CoV-2 virus could all be faithfully detected by the sensor with LOD values of 1 fg/mL (in phosphate-buffered saline), 100 fg/mL (in a clinical transport medium), 1.6 × 10 pfu/mL (in culture medium), and 2.42 × 10^2^ copies/mL (in clinical samples).

Ali et al. [38] developed 3D gold micropillar array electrodes by nano-printing and coating the surface with reduced graphene oxide (rGO) nanoflakes for the immobilization of specific viral antigens through surface functionalization. The electrode was then employed in a conventional electrochemical cell after being integrated with a microfluidic device, and the result could be viewed using a smartphone-based user interface. During the sensing experiment, the introduced antibodies of the SARS-CoV-2 spike S1 protein were attached to the antigens, causing the impedance of the circuit to change and subsequently being detected by impedance spectroscopy with a LOD of 2.8 × 10^−15^ M. In a similar work, El-Said et al. [39] coated the indium tin oxide (ITO) electrode with Au nanoparticle-decorated porous rGO and immobilized anti-COVID-19 antibodies on the surface of the modified electrode. The electrochemical conductivity was improved by the introduction of Au nanoparticles and porous graphene. Finally, the developed biosensor was used to identify the COVID-19 protein with a LOD of 39.5 fmol/L in the concentration range of 100 nmol/L to 500 fmol/L. Wang et al. [40] fabricated a microfluidic immunoassay biochip employing graphene oxide quantum dots to carry out rapid and reliable simultaneous detection of numerous SARS-CoV-2 antigens and antibodies with high throughput and large sensitivity. The platform combined the benefits of nanomaterials to conjugate high-density biomolecules and microfluidics to create a highly efficient biosensing platform that can operate with minimal sample volume, and the resulting LOD was ~0.3 pg/mL. A graphene-based electrical electrochemical vertical device (EEVD) was created by Mattioli et al. [41] for the serologic detection of COVID-19. To properly adsorb AuNP/RBD bioconjugate onto the device interface, non-covalent functionalization with poly-neutral red was used. The operation of the EEVD was based on the high charge carrier mobility of the graphene basal plane. They used immobilized SARS-CoV-2 RBD bioconjugates to detect IgG antibodies based on antigen-antibody interactions with a LOD of 1.0 pg/mL by monitoring the fluctuation of the device’s surface’s interfacial potential. The reduced graphene oxide (rGO) FET, based on Si, was fabricated by Krishna et al. [42] to detect SARS-CoV-2. SARS-CoV-2 monoclonal antibodies were used to functionalize rGO FET to achieve a LOD of 0.002 fM. For label-free and rapid detection of the receptor-binding domain (RBD), nucleocapsid (N), spike (S), and viral particles of SARS-CoV-2 and its variations in saliva samples, Ban et al. [43] created a DNA aptamer-conjugated GFET analytical readout device with extreme sensitivity. For the S protein and N protein of SARS-CoV-2, the sensor’s LOD was 1.28 (PFU)/mL and 1.45 PFU/mL, respectively. Using this portable wireless graphene biosensor, one may quickly (≤20 min) screen for the presence of unprocessed SARS-CoV-2 and its variations in saliva. For the quick and accurate detection of the SARS-CoV-2 Spike S1 antigen, Shahdeo et al. [44] created a technique based on GFET. Using carbodiimide chemistry, the in-house created anti-spike S1 antibody was covalently attached to the surface of a graphene channel having carboxy functionalization. The response of the biosensor was assessed by real-time monitoring of the resistance change caused by antigen-antibody interaction with a LOD value of 10 fM. For the purpose of detecting SARS-CoV-2 infection using nasal swab specimens, Garrote et al. [45] created an AC GFET that acted as a reagent-less quantum-rate-based electrochemical biosensing platform. According to the quantum-rate model, the frequency of electron transport inside molecular structures, as measured in graphene that has biological receptors, may be employed as a transducer signal at the interface with attomole-level sensitivity for biosensing. The quantum-rate technique was employed to integrate the interface response for identifying the S and N proteins of SARS-CoV-2 viral samples with 80% sensitivity, 77% specificity, and a LOD of 0.167 ag/mL. Kadadou et al. [46] deposited rGO on a Si substrate and immobilized SARS-CoV-2 S1 antibodies on the rGO surface using linker molecules for detecting SARS-CoV-2 in municipal wastewater as well as in real clinical samples. The biosensor could detect S1 protein in PBS with a LOD of 0.5 fg/mL and an average reaction time of 240 ms. To identify SARS-CoV-2 visually using the colored LAMP response, Aman successfully devised and made a paper-based analytical device that was integrated with an rGO/MWCNTs heater. For optical detection of the SARS-CoV-2 via colored LAMP reaction, Li et al. [47] successfully built and made a paper-based analytical apparatus combined with an rGO/MWCNTs heater (HiPAD). The HiPAD’s SARS-CoV-2 detection range was 25 to 2.5 10^10^ copies/mL, with 25 copies/mL-1 serving as the LOD value. Furthermore, the single-chamber HiPAD may be readily upgraded to a multiplex HiPAD for multiplex detection with minor modifications. Using the IgG anti-SARS-CoV-2 spike antibody, Ehsan et al. [48] created a paper-based, portable electrochemical impedance biosensor for the detection of the SARS-CoV-2 spike protein. SPEs were used in this label-free platform, which relies on the concept of a probe’s redox reaction impedance, to detect both virus samples obtained from the universal transport medium and antigen spikes residing in nasopharyngeal fluid with a LOD of 0.25 fg/mL. This was accomplished by using high-conductivity graphene/carbon ink with low background impedance and a large dynamic range of detection. Furthermore, it was observed that, compared to chemical immobilization, biological immobilization of antibodies can improve the antibody loading along with the operating range and hence the sensitivity. A GFET was created to detect the SARS-CoV-2 nucleocapsid protein by Novodchuk et al. [49]. The biosensing platform used a graphene oxide gel transducer that was co-doped with boron and nitrogen and functionalized with nucleoprotein antibodies. With a LOD of 10 ag/mL and a broad linear detection range from 10 ag/mL to 1 g/mL, this biosensor was able to identify the viral protein in less than 4 min. Table 1 summarizes the previous discussion on the creation of carbon nanomaterial-based biosensors to identify the SARS-CoV-2 virus.

### 2.2. Carbon Nanomaterials in Personal Protection to Restrict SARS-CoV-2 Transmission

**Figure 4:**
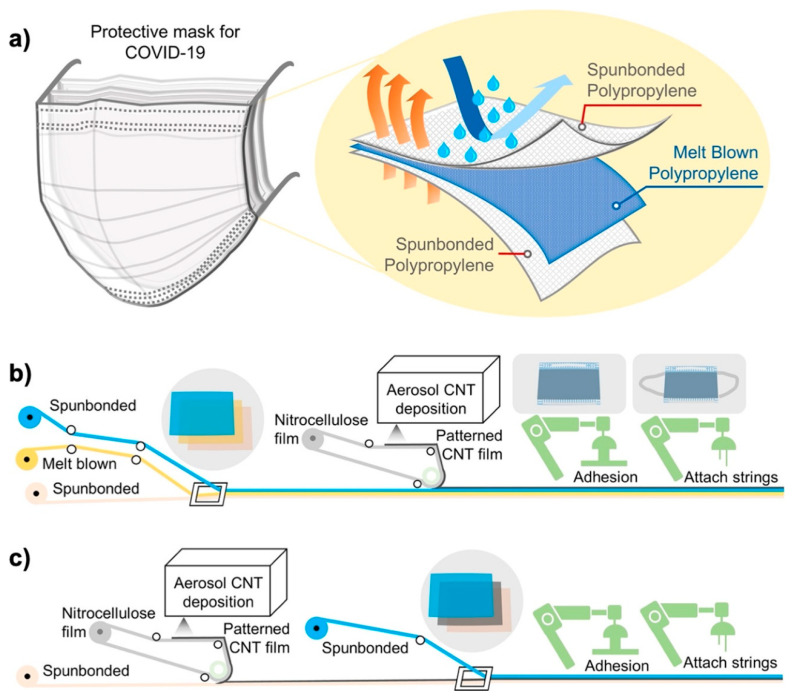
Illustration of (**a**) a conventional face mask with an SMS filter, (**b**) the manufacturing of SMS filters when the CNT filter is added to the process, and (**c**) the manufacturing of face masks where the CNT filter replaces the melt-blown layer. (Reproduced with permission from Ref. [50]).

Aerosol particles and respiratory spores are the most frequent modes of transmission of COVID-19, and thus face masks were marked as an indispensable thing for saving human lives. Lee et al. [50] prepared a CNT-based facemask using a low-cost, facile aerosol-assisted process and found that, owing to the strong, uniform, and durable hydrophobic nature of the CNTs, the CNT mask put up a greater barrier against SARS-CoV-2 than the conventional mask (Figure 4). The large thermal conductivity of CNT facilitates the hyperthermal sterilization capability, providing greater protection against the coronavirus. It was further observed that although the pore size of a CNT mask network was smaller than that of a typical coronavirus, it had the same level of breathability as a polypropylene filter.

**Figure 5:**
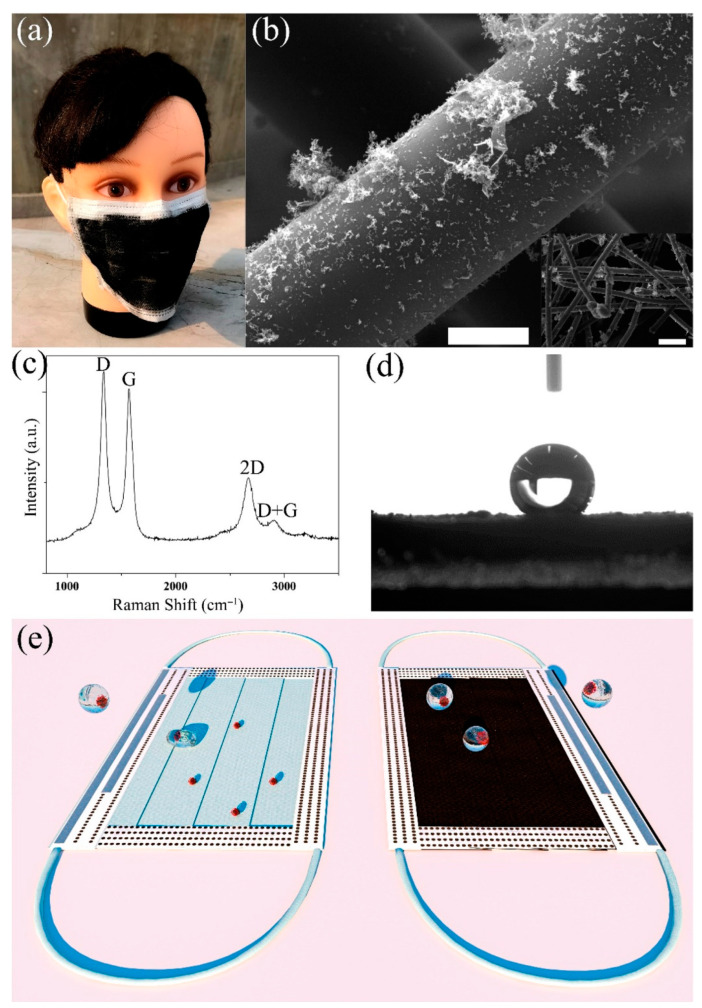
(**a**) Optical image of the laser-fabricated graphene mask. (**b**) SEM of the graphene-coated nonwoven fiber within the surgical mask; scale bar is 10 μm. The inset is a zoom-out image, with the scale bar at 100 μm. (**c**) Raman spectrum of the graphene-coated mask. (**d**) Water contact angle on the graphene-coated mask. (**e**) Illustration of the self-cleaning properties of the black graphene-coated mask (**right**), compared to the pristine blue mask (**left**). (Reproduced with permission from Ref. [57]).

Soni et al. [51] also prepared a CNT mask using a single-step spray-coating technique on a melt-blown polypropylene surgical mask. The prepared mask demonstrated superhydrophobicity and excellent self-sterilization (photothermal) performance. Under solar light, the CNT-coated mask’s virucidal effectiveness was measured to be over 99%. A theoretical study by Patel et al. [52] on binding interactions between CNTs having different morphologies and SARS-CoV-2 revealed that CNTs possess significant binding affinities and strongly interact with the active sites of the coronavirus. The best contender for incorporation in PPE kits to accumulate any free SARS-CoV-2 particles present in the air was determined to be an armchair-type CNT with a hollow core. Singh et al. [53] used a poly(vinylidene fluoride) (PVDF)-CD composite to develop recyclable, self-sterilized (upon short solar irradiation) face masks for curbing the spread of coronavirus. The hydrophobic nature of the composite restricted the accumulation of moisture, while breathability and filtration were ensured through the compact nanopore network. A needleless electrospinning/spraying netting technique was developed by Xiong et al. [54] to fabricate multi-scale nanofiber (NF)/CNT networks, allowing for the layer-by-layer welding of well-dispersed CNT networks on charged NF scaffolds. The improved NF/CNT networks displayed high filtration efficiency and low resistance due to their fluffy structure, narrowly distributed tiny pores, “free molecular flow” behavior, and electrostatic adsorption capability. Their distinctive nanoarchitecture also enabled them to reliably and quickly self-sterilize via photothermal (>99.986% in 5 min) and electrothermal (>99.9999% in 2 min) mechanisms. The most significant benefit is that abandoned NF/CNT filters may be completely recycled into high-efficiency solar vapor generators to desalinate seawater. Wang et al. [55] developed a non-contact electronic skin employing a CNT/PVA nanocomposite that, through the weak spatial field, could react to the finger’s proximity between 0 and 20 mm. After receiving a hydrophobic treatment, the MCP E-skin was not harmed by the scratches caused by the fruit knife and can withstand chemical corrosion. Human-machine interactions in public spaces can use this non-contact sensing to inhibit pathogens. Goswami et al. [56] coated the surface of a face mask (made of polypropylene fabric) using functionalized graphene and graphene nanosheets via a dip coating method to enhance the adsorption capacity of the surface. For the prepared mask with 1.10 mbar of breathing resistance, the values of bacterial filtering efficiency with 20 mm, 10 mm, and a melt-blown filter were 84.0%, 92.61%, and 98.20%, respectively. A laser-assisted transfer method was adopted by Zhong et al. [57] to deposit graphene over the surface of nonwoven masks to make the outer surface superhydrophobic (Figure 5). The superhydrophobicity of the mask surface was capable of repelling incoming aqueous droplets. Moreover, the mask can be sterilized through the photothermal action of sunlight to make it reusable.

Graphene nanosheet-embedded carbon (GNEC) film was prepared by Lin et al. [58] using an electron cyclotron resonance (ECR) sputtering system and employed the same to fabricate a face mask through the ultrasonic extrusion method. The prepared mask displayed exceptional properties such as superhydrophobicity, excellent bacterial filtration efficiency, and self-photo-sterilizability. The experimental study by Maio et al. [59] revealed that by incorporating graphene nanoplatelets into polylactic acid filaments (PLA-G), it is possible to create 3D-printed objects that can be sterilized using sunlight-like near-infrared light exposure. SARS-CoV-2 virus particles on the surface of 3D printed PLA-G were killed using this technique after 3 min of exposure. Due to its excellent biocompatibility, 3D-printed PLA-G can serve as the perfect material for creating sterile daily products and protective gear that are meant for many users. An inexpensive electrothermal mask with exceptional self-sterilization capabilities was developed by Shan et al. [60]. A filter layer comprised of melt-blown nonwoven fabrics (MNF) was first covered with flexible and conductive cloth tape to create interdigital electrodes. Next, a graphene layer with superior thermal and electric conductivity was deposited on the MNF. The graphene-modified MNF (mod-MNF) could produce appreciable amounts of heat to reach a temperature above 80 °C at a voltage of only 3 V, and the generated heat could destroy most of the viruses that were bound to the filter layer and mask surface. Furthermore, the electrothermal masks could retain almost the same PM elimination efficacy after being electrified 10 times, indicating their exceptional reusability. An inexpensive, triboelectric nanogenerator (TENG)-assisted, 3-layer mask was designed by Figerez et al. [61], in which the polypropylene layer was modified with GO and a polyvinylidene fluoride mixture to achieve high filtration efficiency for 300-nm-sized particles. The modification step improved the amount and retention capacity of both the voltage and charge of the polypropylene layer. The triboelectric rechargeability was employed for sanitization and could be realized through small mechanical agitations such as hand-bending.

### 2.3. Carbon Nanomaterials as Antiviral Agents for Removal of the SARS-CoV-2

**Figure 6:**
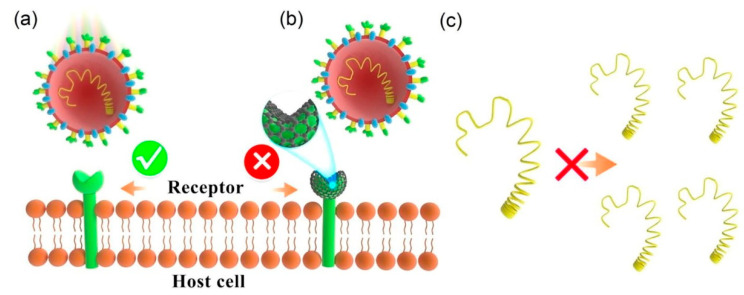
The schematic representation concerning the antiviral activity of functionalized graphene QDs. (**a**) The binding interaction between the S protein of the virus (HCoV-229E) and the host cell receptor causes viral diseases. (**b**) Such binding can be inhibited by the presence of ODs. (**c**) This mechanism can lead to the inhibition of viral genome replication. (Reproduced with permission from Ref. [64]).

All of the carbon nanomaterials established themselves as effective antiviral agents (Figure 6) against a broad spectrum of viruses [62,63]. Thus, all the carbon nanomaterials were extensively probed by researchers around the globe to find out their effective inhibition capacity against the novel coronavirus.

A few theoretical studies were carried out using density functional theory [65], docking and molecular dynamics [66], and energy decomposition techniques [67] to check the feasibility of using fullerene for inhibiting the coronavirus. The results revealed that, because of its hydrophobicity and stiffness, fullerene could minimize the factors that often oppose its binding with the SARS-CoV-2 virus, which makes fullerenes an excellent inhibitor. Even metal-doped fullerene complexes can be employed as potential drug delivery materials to combat COVID-19 [68]. Hurmach et al. [69] assessed the anti-coronavirus activity of water-soluble pristine C_60_ fullerene using theoretical modeling (molecular dynamics and docking methods) as well as in vitro systems. The results showed that to get coupled with SARS-CoV-2 proteins, fullerene filled the binding pocket of the virus and became locked there through stacking and steric interactions. This led to the formation of stable complexes, which in turn impeded the virus’s ability to function. As in the case of fullerene, scientists also performed some theoretical studies using DFT [70] and all-atom molecular dynamics simulation [71] to estimate the potential of CNT as an antiviral agent against SARS-CoV-2. The studies showed that the CNTs could be used for drug delivery as well as an inhibitor to combat COVID-19. It was further observed that CNTs can be adsorbed on the surface of the spike protein of SARS-CoV-2 and affect its stability by altering the structure. Kalkal et al. [72] hypothesized that CDs extracted from allium sativum (AS) could be used as an effective theranostic agent that was competent in reducing the progressive symptoms of COVID-19 by improving respiratory functionalities. Pramanik et al. [73] showed that graphene quantum dots (GQD) conjugated with a human host defense neutrophil and a human cathelicidin peptide were capable of preventing the delta variant of SARS-CoV-2. It has been claimed by Huang et al. [74] that the novel coronavirus can be effectively neutralized by laser-induced graphene (LIG) without the addition of any metals. The authors also testified that the enhanced inactivation capability was a result of a synergistic interaction between the photothermal effect and the hydrophobicity of the LIG. They also reported that [75] LIG has improved antibacterial capability against aerosolized microorganisms. The inhibition rate rose to about 81% when LIG was used. Within 10 min, 99.998% bacterial killing efficiency was achieved when combined with the photothermal effect. Graphene can also be utilized as a broad-spectrum virus inhibitor, as reported by Donskyi et al. [76]. They functionalized graphene with defined dual polyglycerol sulfate/aliphatic chains for the disruption of the coronavirus envelope, and the length of the chains played a crucial role in virus inhibition. Unal et al. [77] employed theoretical as well as practical approaches to examine the inhibitory properties of GO against SARS-CoV-2 surface proteins and cell receptors. The study revealed that the copies of three separate viral clades’ were drastically decreased by using thin, biological-grade GO nanosheets. This research showed that, despite the existence of any alterations on the viral spike, GO nanosheets can interact with SARS-CoV-2 surface components and interfere with their infectivity.

### 2.4. Carbon Nanomaterials in Making Vaccines for SARS-CoV-2

**Figure 7:**
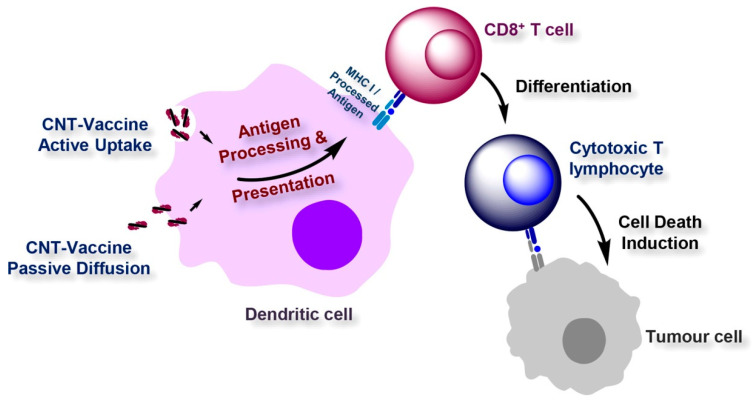
Proposed pathways for MHC presentation of CNT-delivered antigens. CNTs could deliver the incorporated antigen to the cytosol of the DCs via two proposed routes, where degradation by the proteasome and subsequent MHC class I presentation occur. The ability of CNT-antigen conjugates to passively diffuse through the cell membrane could directly deliver the loaded antigen to the cytosol. Alternatively, following the active uptake of CNT-antigen conjugates by DCs and the subsequent endosomal escape, the incorporated antigen could gain entry into the cytosol. In addition, to overcome the need for endosomal escape, antigenic fragments yielded from antigens processed in the endosomes by endosomal proteases could be loaded onto the MHC class I molecules recycled from the plasma membrane. Furthermore, professional cross-priming DCs could translocate the endocytosed antigenic cargo to the MHC class I pathway via the utilization of a cross-presentation mechanism. Lysosomal degradation of CNT-delivered antigens that fail to escape the endosomes could be followed by MHC class II presentation. (Reproduced with permission from Ref. [78]).

Among the different allotropes of carbon, CNT [78] and graphene [79] were widely used to develop vaccines for various diseases (Figure 7).

In 2020, a Chinese group from the Shanghai National Engineering Research Center for Nanotechnology published a report [80] and later filed a patent [81] about the application of GO as a carrier for a nano-coronavirus recombinant vaccine and claimed that the vaccine could be used for the prevention and treatment of the novel coronavirus. Zhou et al. [82] experimented to increase the efficacy of dendritic cell (DC) vaccines through the introduction of GO nanosheets of varied sizes. The study revealed that GOs with diameters greater than 1 µm adhere strongly to the DC surface and act as a “nanozipper”, resulting in the formation of a stable microenvironment for T cell activation. Moreover, in mice infected with a SARS-CoV-2 strain, GO-adjuvanted DCs stimulated cytotoxic T-cell immune responses targeting SARS-CoV-2 spike 1 and cleared more than 99.7% viral RNA.

## 3. Challenges and Future Scope

The several routes through which carbon nanomaterials can be employed to suppress new coronaviruses are discussed. Carbon nanomaterials with diverse structural and behavioral properties were identified to have enormous promise for combating COVID-19 in a variety of ways, including biosensing elements, a component of PPE and masks, antiviral medicine [83], and vaccine ingredients. However, due to the existence of atomic-scale characteristics such as defects, impurities, disorders, grain boundaries, and so on, large-scale growth of carbon nanomaterials with reproducibility and a well-defined structure is problematic. A minor modification in the nanostructure can cause significant changes in its properties, resulting in a variation in the outcome when employed in the medical field. In the case of bio-sensing, it can be stated that, till now, the reports belong to the initial phases of sensing experiments, and with time, the sensor capability deteriorates owing to the degradation of the contacts, which needs to be measured, and remedial measures must be taken for faithful detection [84]. Biocompatibility of carbon nanomaterials is another key concern during in vivo testing; hence, various research is being undertaken across the world to precisely analyze the toxicity of carbon nanomaterials [85,86,87]. Furthermore, organizations must carefully assess the risk aspects of any carbon nanomaterials in terms of toxicity, and suitable legislation must be enacted to ensure their safe deployment in relevant industries and healthcare sectors. The majority of the inhibitory properties have been documented from lab-based investigations, and commercialization is necessary to provide advantages to the general public. Along with biocompatibility, process cost will be a key challenge in the transition from lab to market, which must be carefully addressed. It must be remembered that thousands of tons of medical waste, including used disposable masks, PPES, vaccination vials, and injection syringes, are produced globally each day, placing a heavy burden on the environment. Thus, some pathways [88] have to be devised to comply with the motto of the 3Rs, (Reduce, Reuse, and Recycle) to save the environment. In summary, although there are many obstructions to accomplishing the successful and inexpensive application of carbon nanomaterials against SARS-CoV-2 while maintaining a low-toxicity level, it can be projected that carbon nanomaterials will overcome all barriers in the near future by using their enormous potential.

## Figures and Tables

**Table 1:** Carbon nanomaterials-based biosensors to identify the SARS-CoV-2 virus.

Carbon Nanomaterial	Target	Sensor Type	Limit of Detection (LOD)	Detection Range	Detection Platform	Ref.
Carbon Dot	IgM and IgG	Optical	100 pg/ml	100 pg/mL to 100 μg/mL	Surface of carbon dot	[19]
	RNA	Optical	81 copies/μL.	NA	Surface of carbon dot	[20]
	Nucleocapsid proteins	Optical	10 pg/mL	10 pg/mL to 1 μg/mL	Surface of carbon dot	[21]
Carbon Nanotube	Nucleocapsid proteins	Immunosensor	5.62 fg/mL	NA	CNT thin-film	[22]
	Spike protein (S1)	FET-based	4.12 fg/mL	4.12 fg/mL to 5.0 pg/mL	CNT surface	[24]
	RNA	FET-based	10 fM	NA	CNT surface	[25]
	Spike antigen (SAg) and Nucleocapsid antigen (NAg)	FET-based	0.55 fg/mL for SAg and 0.016 fg/mL for NAg	0.55 fg/mL to 5.5 pg/mL for SAg and 16 fg/mL to 16 pg/mL for NAg	CNT surface	[26]
	Spike protein	Optical	12.6 nM (receptor-binding-domain RBD)	NA	SWCNT substrate	[29]
	Antibodies to Spike protein	Electrochemical	0.7 pg/mL	0.7 pg/mL to 10.0 ng/mL	Electrode	[31]
	Spike protein S1	Electrochemical	7 nM	NA	Electrode	[32]
	Spike protein	Immunosensor	350 genome equivalents/mL	NA	Electrode	[33]
	DNA	Electrochemical	aM level (for impedimetric) and zM level (for amperometric)	1.0 × 10^−18^ M to 1.0 × 10^−11^ M	Electrode	[34]
	Whole Virus	Electrochemical	57 pg/mL	NA	Electrode	[35]
Graphene	Spike protein	FET-based	1 fg/mL (in phosphate-buffered saline) and 100 fg/mL (in a clinical transport medium) and 1.6 × 10 pfu/mL (in culture medium) and 2.42 × 10^2^ copies/mL (in clinical samples)	NA	Graphene surface	[37]
	Antibodies to Spike protein S1	Electrochemical	2.8 × 10^−15^ M	NA	Electrode	[38]
	Spike protein S1	Electrochemical	39.5 fM	39.5 nM/L to 500 fM	Electrode	[39]
	Spike antigen (SAg) and Nucleocapsid antigen (NAg)	Immunosensor	0.3 pg/mL.	0.3 to 10^3^ pg/mL	Graphene oxide quantum dots surface	[40]
	IgG	Immunosensor	1.0 pg/mL	10^−12^ to 10^−7^ g/mL	Graphene surface	[41]
	Spike protein	FET-based	0.002 fM	NA	rGO surface	[42]
	Spike (S) protein and Nucleocapsid (N) protein	FET-based	1.28 (PFU)/mL (for S protein) and 1.45 PFU/mL (for N protein)	100 fM to 100 nM	Graphene surface	[43]
	Spike S1 antigen	FET-based	10 fM	10 fM to 1 μM	Graphene surface	[44]
	Spike (S) protein and Nucleocapsid (N) protein	FET-based	0.167 ag/mL	NA	Graphene surface	[45]
	Spike antibody	Immunosensor	2.91 copies/mL (Synthetic) 0.5 fg/mL (raw wastewater)		Graphene surface	[46]
	N-Gene	Optical	25 copies/mL	25 to 2.5 × 10^10^ copies/mL	NA	[47]
	Spike protein	Electrochemical	0.25 fg/mL	0.25 fg/mL to 1 µg/mL	Electrode	[48]
	Nucleocapsid protein	FET-based	10 ag/mL	10 ag/mL to 1 g/mL	Graphene surface	[49]

## Data Availability

Not applicable.
